# The feasibility of implementing an English language version of GLA:D Back

**DOI:** 10.1186/s40814-020-00758-z

**Published:** 2021-02-01

**Authors:** J. Lemieux, G. Kawchuk, A. Kongsted, J. Hartvigsen, V. Abdollah, A. Jones

**Affiliations:** 1 Department of Physical Therapy, Faculty of Rehabilitation Medicine, University of Alberta, Edmonton, Canada; 2 Department of Sports Science and Clinical Biomechanics, University of Southern Denmark, Odense, Denmark

## Abstract

**Background:**

Evidenced-based clinical guidelines for the treatment of low back pain (LBP) consistently suggest educating patients about their back pain, its natural course, and providing advice to keep active and continue working. Despite this evidence, clinicians routinely do not follow these recommendations resulting in ineffective and fragmented care. GLA:D® Back, a standardized care package, was originally developed in Denmark to assist clinicians in implementing evidence-based care. This study will evaluate the feasibility of implementing the English version of the Danish GLA:D® Back program in Alberta, Canada.

**Methods:**

Thirty-five clinicians from nineteen clinics in Alberta, Canada, participated. Feasibility of program implementation, our primary objective, was evaluated within 3 months. Feasibility success was defined as 50% clinician/clinic adoption in addition to 66–88 enrolled participants registered in the database. Our secondary objectives included collecting data pertaining to clinician confidence, attitudes and behaviour of treating patients, perceived barriers and facilitators of program in addition to collecting patient-data regarding pain, function, general health and self-efficacy.

**Results:**

The majority of the clinics (15/19, 79%) offered GLA:D® Back to their patients within the study period. Of the participating clinicians, GLA:D® Back was delivered by (25/35, 71%) of clinicians. In total, 78 patients were enrolled in the program and (69/78, 88%) participants attended the final assessment. Secondarily, clinicians demonstrated a biomedical and behavioural orientation along with high confidence when treating LBP patients while patient outcomes trended toward improvement.

**Conclusion:**

The English translation of the Danish GLA:D Back program was feasible for Albertan clinicians to implement into practice in both urban and rural settings.

## Key messages regarding feasibility

1. What uncertainties existed regarding the feasibility?
  Is the English version of GLA:D® Back feasible when taught and tested in English?
  Can clinicians trained in GLA:D® Back successfully implement the program?
2. What are the key feasibility findings?
  The majority of clinicians trained in GLA:D® Back employed the program in clinical practice.
  Participating clinicians had positive impressions of the program.
  Clinicians’ ratings of program content, usefulness and novelty were high.
  Clinicians were satisfied with the translated materials and the program itself.
3. What are the implications of the feasibility findings for the design of the main study?
  Training materials translated from Danish to English can be used to successfully train English-speaking clinicians.
  Trained clinicians can successfully implement GLA:D® Back in practice.
  Patient recruitment was difficult in shift workers or those with insufficient resources and/or insurance coverage.

## Introduction

Low back pain (LBP) is a common, chronic recurrent symptom that is responsible for more years lived with disability than any other condition worldwide [1]. As a result, the societal, health care and economic burdens associated with LBP are equal to or greater than those of other, high-cost conditions such as cancer, cardiovascular disease, autoimmune diseases and mental health [2].

Evidence-based clinical guidelines for the treatment of LBP consistently suggest educating patients about what back pain is, its natural course, and giving advice about staying active and at work [3]. In addition, most of these guidelines recommend supervised exercise, manual therapy alone or in combination with exercise and discourage routine imaging, administration of opioids and reserve surgery for a few with specific indications [4]. Regardless, clinicians of various professions remain unclear about how to manage LBP [5] as evidenced by their ongoing use of treatments and procedures not recommended by the guidelines themselves [6, 7] which often results in ineffective and fragmented care [8–10].

Standardized care packages based on guideline recommendations are suggested as a tool to assist clinicians in implementing evidence into clinical practice [11]. One such program is Good Life with Osteoarthritis in Denmark (GLA:D®) for people with knee or hip pain. The GLA:D® program, described in detail elsewhere [11], is a standardized program that consists of group-based patient education together with 6 weeks of twice weekly supervised group exercise while patient outcomes are collected systematically in a clinical registry [11]. Between 2013 and 2017, more than 1100 trained clinicians have entered 30,000 patients in the GLA:D® knee and hip registry in Denmark alone [12]. This program has made standardized evidence-based care widely available and has been successful in a variety of ways including reducing disability, pain and medication use [13]. GLA:D® knee and hip is now available in Canada, Australia, Switzerland and New Zealand [13].

Based on the success of this approach, GLA:D® Back was created to address people seeking care for persistent or recurrent back pain with the goal of promoting self-management and patient empowerment. The GLA:D® Back program maintains the same core components of patient education, supervised group exercise classes and a registry to record patient and clinician outcomes throughout the program. The program itself is taught to clinicians from approved professions (presently chiropractic and physiotherapy) in a 2-day seminar format which then qualifies attendees to offer to program in their community.

To date, the GLA:D® Back program has been launched in Denmark with 619 clinicians trained and approximately 2800 patients registered (April 2018–December 2019). Early indications from Danish pilot data suggest that GLA:D® Back is capable of reducing disability, pain and medication use while increasing physical capacities [14]. To build on this success internationally, it was necessary to translate the GLA:D® Back program materials into English and evaluate the success of this translation in an English setting.

This paper reports on a feasibility study where the Danish GLA:D® Back program was translated into English and then subsequently delivered in private physiotherapy and chiropractic clinics in Alberta, Canada. Although cultural, professional and legislative differences may exist between implementation of the program in Denmark versus an English-speaking country, we hypothesize our results would be similar to those described in the Danish pilot [14].

The primary objective of this feasibility study was the following:

1. To evaluate the adoption of GLA:D® Back in clinical practice among Canadian clinicians volunteering to the feasibility study.

In addition, our secondary objectives included the following:

1. To evaluate clinician perception of GLA:D® Back training and GLA:D® Back implementation.
2. To evaluate the potential change in clinicians’ beliefs and behaviours about back pain after completion of the program.
3. To describe the patient participants characteristics who enrolled in GLA:D® Back as well as patient self-reported outcomes related to function, pain, general health and self-efficacy.

## Methods

### Overview

**Fig. 1:**
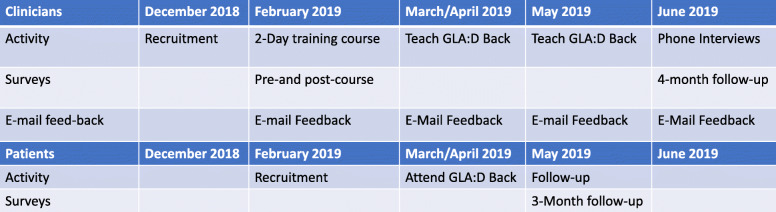
Study Flow. Overview of activities and data collections at the level of the clinicians and patients. Please refer to the text for exact time periods.

The feasibility study of the GLA:D® Back program was overseen at the University of Alberta and implemented by trained community clinicians based in urban- and rural-based physiotherapy and chiropractic clinics in Alberta. Pre-implementation training of clinicians occurred over a 2-day training course at the University of Alberta. Following training, clinicians delivered GLA:D® Back at their clinics on a voluntary basis. During the course of the study, clinician and patient’s data were collected at baseline and at subsequent intervals via electronic questionnaires administered through Research Electronic Data Capture (REDCap). This study was approved by the Human Research Ethics Board of the University of Alberta (Pro00085118). GLA:D® is a non-profit initiative whose name is trademarked by the University of Southern Denmark (SDU). For an overview of the study’s events and chronology, please refer to Fig. 1.

### Clinician education

Clinicians were recruited to the study using content approved for distribution on the Alberta College and Association of Chiropractors website (albertachiro.com), and the Physiotherapy Alberta College + Association website (physiotherapyalberta.ca), as well as through personal contacts.

All participating clinicians provided their consent for data to be collected and used for research purposes prior to training. On February 16–17, 2019, enrolled clinicians participated in the 2-day course. The course was taught by developers of GLA:D® Back and adapted from the Danish training program, which consisted of a mixture of lectures and practical workshops aimed to develop the clinicians’ ability to deliver the program. Goal setting, clinical tests, patient education, supervised exercises and data registration were introduced during the training workshop [12]. Role-playing and skills’ training were used to familiarize clinicians with the educational component, performance-based tests and exercises. Participants worked in groups to practice delivering key messages from the education content as they would in a real patient education session. Upon completion of the course, participants were given access to the REDCap data registry which also acted as a repository. Materials included standardized patient education sessions (PowerPoint with manuscript, exercises to support patients’ reflections, two posters with patient education key messages), exercise programs, content for patients and primary care physicians about the feasibility project itself.

### Patients

Patients were recruited directly by trained clinicians within the boundaries set by their respective provincial regulatory bodies. Adult patients were eligible for the GLA:D® Back program if presenting with persistent or recurrent low back pain without or with leg pain with no known specific pathology and a perceived need for improved self-management skills. Execution of inclusion and exclusion criteria as well as collection of informed, written consent was performed by the clinician. Patients were required to pay $100 CAN for the initial assessment and $30 for subsequent sessions for a total of 20 sessions totaling $640 to complete the full program. Patients’ fees for three of the rural clinics were fully subsidized by the provincial health care program (Alberta Health Services).

### The GLA:D® Back intervention

GLA:D® Back is designed to assist and promote patient self-management and self-efficacy by providing knowledge of pain mechanisms, reducing fear of movement and supporting patients in gaining control of pain, function and also to promote physical activity and exercise [12]. In brief, an individual assessment provided by the trained GLA:D® clinician is completed to determine patient eligibility. If eligible, four clinical tests are then performed: standing forward bending test [15, 16], trunk flexor endurance test [17, 18], back extensor endurance test [17–19] and sit to stand in 30 s [20, 21]. Personal goals (S.M.A.R.T. rehabilitation goal setting) [22] are discussed then established, and the starting level of 8 separate groups of exercises is determined [12]. The participant is then scheduled for two 1-h group-based education classes and bi-weekly 1-h supervised group exercise sessions for 8 weeks [12]. Group size was recommended by the GLA:D® developers to be 4–8 participants with a maximum group size of 10 [12]. The program ends with a final assessment where personal goals are revisited, and the four clinical tests are repeated. A more detailed description of the theory and development of GLA:D® Back has been published previously [12].

GLA:D® Back was developed around social cognitive theory and cognitive behavioural theory [12]. As such, education and movement (exercises) are used to support the promotion of self-efficacy [12]. Key messages in the patient education, (i.e. LBP is common, pain intensity does not reflect tissue injury and the spine is strong and designed for movement) are displayed throughout the education sessions and these messages are further incorporated into the exercise sessions one per week (8 key messages total) [23]. Throughout the group exercise sessions, the participants’ existing beliefs and concerns are discussed.

The exercise section of GLA:D® Back incorporates strength, endurance and flexibility training divided into eight groups with four levels of difficulty in each [12]. The starting level for each exercise is agreed on by the GLA:D® Back clinician and the patient as suitable for the participant’s tolerance [14]. Participants are encouraged throughout the exercise sessions to explore a variety of movements rather than doing the exercise in one “correct” way [12]. Participants self-progress under guidance to more difficult exercises throughout the hour sessions, while clinicians guide the performance of exercises and the choice of exercise level to the degree needed [12]. Thus, the exercise program is both standardised and highly individualised.

### Feasibility outcomes

Four months after training, clinicians were asked the following questions: When did you start offering GLA:D® Back in the clinic? How many cohorts have you started? What is the number of patients in the registry? Based on the GLA:D® Back 2019 Danish Annual Report [24], our criteria for feasibility success was pre-defined as 50% of clinics/clinicians conducting the program within 3 months of completion of the training course resulting in 66–88 participants registered in the database. During the study period, clinicians provided the investigators with ongoing, informal feedback via a private smart app channel as well as asking for help or information on any aspect of the GLA:D® Back program. Clinicians were also free to express concerns or ask questions of the investigators via e-mail.

### Clinician outcomes

Clinicians were surveyed at three-time points: 1 week prior to participating in the 2-day GLA:D® Back workshop immediately after the GLA:D® Back training course, and 4 months after completing the GLA:D® Back training course.

Specifically, the pre-course survey included closed-ended questions about clinician demographics (age, sex, profession, years of clinical experience, role at clinic, i.e. clinic owner, self-employed, employee, previous experience with GLA:D® knee/hip) as well as an assessment of their confidence with handling back pain patients (Practitioner Confidence Scale-PCS) and their attitudes and beliefs about back pain (Pain Attitudes and Beliefs Scale-PABS). The PCS and PABS were repeated by an electronic survey 4 months following the training course. The PCS is a 4-item scale measuring confidence in managing people with back pain [25]. Each item is scored on a 5-point scale. (1 = “strongly agree” to 5 = “strongly disagree”) with the resulting sum score ranging between 4 and 20 where a higher score indicates a lower confidence [25]. The PABS is used to assess the predominance of two treatment orientations toward the management of back pain: biomedical orientation or behavioural orientation [26, 27]. The biomedical subscale consists of 10 items (sum score 10–60) and the behavioural subscale of 9 items (sum score 9–54). Each item is scored on a 6-point scale. (1 = “totally disagree to 6” = “totally agree”. Higher scores reflect a more biomedical or behavioural orientation respectively [26, 27].

Immediately following the GLA:D® course, clinicians were asked to rate the course with respect to course content, novelty and usefulness with each of these domains scored on a scale of 0 to 10 (from 0 = “very poor” to 10 = “very good”). Clinicians were also asked to answer survey questions related to the DIBQ (Determinants of Implementation Behaviour Questionnaire) that were adapted for use in GLA:D® Back (Ris I, Schröder K, Kongsted A, Abbott, A, Nilsen P, Hartvigsen J ÖB. The Determinants of Implementation Behaviour Questionnaire (DIBQ): tailoring to best-practice low back pain primary care program implementation, and pilot testing in Sweden and Denmark. Manuscript submitted).

To better identify and understand barriers and facilitators of implementation, 4 months following the training course, the lead author (J.L.) conducted one-on-one semi-structured telephone interviews with all clinicians. The interview guide was centered on perspectives regarding the content of the clinical intervention and the implementation at their clinic with perspectives on patient recruitment for the program. The telephone interviews were 20 min in duration, audio-recorded, transcribed verbatim and quotes related to various themes of GLA:D® Back were identified by J.L.

### Patient outcomes

Patients who expressed interest in the study voluntarily provided their email address and received detailed information about the study as well as an electronic form to provide consent to be enrolled. If enrolled, they then received an automatically generated link to a baseline survey on the day of the first consultation and 3 months later following completion of the GLA:D® program. If there was no response within 3 days, an automated reminder was sent.

The baseline survey collected demographic information, information on LBP history, previous treatment, and self-reported risk factors for a poor prognosis (The STarT Back Screening Tool) [28].

Both at baseline, and at the 3-month follow-up, a series of patient reported outcome measures were collected including pain intensity via a numerical pain rating scale (0–10 NRS), activity limitation (Oswestry Disability Index (ODI); 0–100), illness perceptions (the Brief Illness Perceptions Questionnaire (B-IPQ); 0–80), fear of movement (Fear Avoidance Beliefs Questionnaire (FABQ); 0–24), quality of life (SF-36 subscales) and “perceived physical fitness” (0–40) via self-assessed strength, endurance, cardiovascular fitness and balance. The use of pain medication was documented as a binary yes/no response.

At 3 months after completion of the program, the above patient-reported outcome measures (PROMS) were collected again in addition to reporting of usage of non GLA:D® interventions (e.g. spinal manipulative therapy, massage therapy). Participants were also asked if they were satisfied with the GLA:D® Back program using a 5-point Likert scale (1- “not at all” to 5- “to a great extent”).

Finally, results for clinical tests conducted before and after GLA:D® Back were included in the participants database. These measures included physical performance assessed by a standing forward bending test (4-no pain with normal movement, 3-pain and normal movement, 2-no pain with abnormal movement, 1-pain and abnormal movement, 0-test not completed), the Ito extensor endurance test (static extension from 0 s minimum up to a maximum of 3 min), the trunk flexor endurance test (0 s up to a maximum of 2 min in static sit-up position) and the sit to stand test (stand number of repetitions of standing from seated in 30 s).

Patient adherence was measured by the number of sessions attended.

### Sample size

In this feasibility study, we limited participation to 20 clinics who each conducted a single GLA:D® Back intake of no more than 10 participants/clinic. Clinician participation was capped to 20 clinics due to the size of our training facility. As for the limit of 10 patients per GLA:D® class, this is the number described as optimal by the GLA:D® Back originators. As such, no formal sample size calculation was performed.

### Analyses

Descriptive statistics were performed for all quantitative data collected.

Feasibility of adoption was based on multiple measures including the (1) number of clinics that offered the GLA:D® Back program and registered participants into the clinical registry within the first 3 months of the feasibility study, (2) number of clinicians that did the same, (3) the total number of participants enrolled in GLA:D® Back in that time period and (4) participants that finished the course with a final exit assessment and (5) the number of completed participants questionnaires at the 3-month follow-up.

Clinicians attitudes and beliefs about back pain were measured by describing group medians, first and third quartiles on the PCS, and the PABS at baseline and 4-month follow-up. To evaluate the with-in clinician change on the PABS, the median change scores were calculated together with first and third quartiles.

Subsequent inferential analyses were carried out in an exploratory manner using R software (Version 3.6). We employed the Wilcoxon signed rank test to evaluate the change in both clinician and participants’ measures as many of these outcomes did not meet established assumptions for parametric testing.

For the qualitative feedback collected through semi-structured interviews, the use of thematic analysis was employed pragmatically by grouping quotes into themes relating to the clinician course, participants’ education, participants’ exercise, participants’ recruitment and the logistics of implementing the program in clinic. All clinicians were accommodating to the procedure and the interview feedback resulted in rich information to improve upon the GLA:D® Back program.

## Results

### Participating clinicians

Thirty-five clinicians (*n*=25 physiotherapists, *n*=10 chiropractors) with varying clinical experience (56% had 11–20 years clinical experience) participated in the 2-day course. All clinicians 100% (35/35) completed the GLA:D® Back pre-course survey and the post-course survey. At 4 months, 77% (27/35) of clinicians completed the follow-up survey. Sixteen clinics were represented by two clinicians and the three rural clinics were represented by one clinician each. Six out of the fifteen clinics were concurrently offering the GLA:D® knee and hip program and 13/35 (36%) of clinicians have referred patients to the GLA:D knee and hip program (Table 1).

**Table 1:** Clinician characteristics and select outcomes

	*n* (%) (unless other specified)
Age, mean (range)	38 (24–58)
Female	15 (42%)
Physiotherapist	25 (71%)
Chiropractor	10 (29%)
Clinic owner	12 (33%)
Self-employed in a clinic own by someone else	11 (31%)
Employee	12 (33%)
Clinical experience	
0–5 years	10 (28%)
6–10 years	6 (17%)
11–20 years	10 (28%)
> 20 years	10 (28%)
**Previous experience with GLA:D® for knee/hip**	
No experience	15 (42%)
Have referred to GLA:D in house	10 (28%)
Have referred to GLA:D in another clinic	3 (8%)
Have instructed GLA:D groups	2 (6%)
**Evaluation of the GLA:D® Back training course, median (range)**	
Content (0–10)	9 (5–10)
Usability (0–10)	9 (5–10)
Novelty (0–10)	9 (1–10)
**Overall impression of the GLA:D Back programme**	
Very good	6 (24%)
Good	15 (60%)
Neither good nor bad	4 (16%)
Bad	0
Very bad	0
**Satisfaction with patient education materials**	
Very satisfied	6 (24%)
Satisfied	19 (76%)
Neither satisfied nor dissatisfied	0
Dissatisfied	0
Very dissatisfied	0

### Clinician demographics at baseline

Clinicians were 42% female, 38 years old on average and were split into clinic owners (33%), self-employed (31%) or employees (33%) at a clinic with (71%) being physiotherapists and (29%) chiropractors. Most clinicians had greater than 11 years of experience (56%) in clinical practice. Six percent (6%) had prior experience teaching GLA:D® knee/hip program, and 58% were familiar with the GLA:D® knee/hip program (Table 1). Clinicians had a moderate biomedical and high behavioural orientation at baseline (PABS) and moderately high confidence when treating patients with back pain at baseline (PCS) (Table 2).

### Patient demographics at baseline

Most participants (60%) had experienced LBP for more than 1-year and had prior treatment for more than a 4-week duration (69%). The average age of patient participants was 56 (SD=13) years old with 66% (*n*=49) being females (Table 3). At the time of enrollment, 50% (*n*=39) of participants were taking over the counter or prescription medications and had slightly higher than moderate B-IPQ scores median difference: 6.5, (*p*=0.015) (Table 4). Almost half of the participants were classified as high risk (33/74, 45%) for risk factors of a poor prognosis according to the STarT Back Tool [28] (Table 3). Participants at baseline scored moderately high for fear avoidance behaviour with a value of 15 (range 0–24) and a median decrease at post-intervention of: 5.0, (*p* ≤ 0.001), and had a medium perception of physical fitness at baseline with a value of 19 (range 0–40) and a median increase of 3 (*p*=0.031) (Table 4). No adverse effects were reported in this study.

**Table 2:** Clinician outcome measures evaluated by Wilcoxon sign test

Variable	Pre-training median	Pre-training Q1, Q3	Post-training median	Post-training Q1, Q3	Difference in median (post-pre)	Pseudo-median	*p*
PCS (4–20)	10.5	10.0, 11.0	9.0	9.0, 10.0	− 1.5	1.5	< 0.001
PABS Biomedical (10–60)	27.0	23.0, 33.5	23.0	18.0, 29.5	-4.0	4.0	0.005
PABS Behavioural (9–54)	39.5	36.5, 42.0	42.0	37.5, 44.0	2.5	-1.50	0.023

Note: *P* values for differences are from Wilcoxon signed-rank. *p* indicates *p* value

*Q1* first quartile, *Q3* third quartile, *PCS* Practitioner Confidence Scale, *PABS* Pain Attitudes Belief Scale

### Feasibility outcomes

The majority of the clinics (15/19, 79%) offered GLA:D® Back to their patients within the study period. Four clinics, three urban clinics and one rural clinic did not start the program because of difficulty recruiting patients from colleagues at their clinics (referrals), a perceived lack of the ideal patient for the program or their clinic was not ready to start the program in the first 3 months following training. GLA:D® Back was delivered by (25/35, 71%) of clinicians. The 10 clinicians who did not deliver the intervention expressed the intention to deliver the program in the future. One clinician who delivered the program reported that they would not offer the program again in the future as “it does not fit with my practice style”. The 15 clinics that actively participated in delivering the program enrolled a total of 78 participants who also attended the initial assessment (range 1–7 participants per group) within the first 3 months after having taken the course. Out of these fifteen clinics, ten clinics had a group size of at least 4 participants. Of these seventy-eight participants, (69/78, 88%) attended the final assessment with nine participants dropping out for various reasons including a change in diagnosis of their condition (i.e. ankylosing spondylitis), worsening of symptoms unrelated to the back (i.e. shoulder), worsening of back symptoms, moved locations or experienced a change in their work schedule. Of the enrolled seventy-eight participants, (52/78, 67%) completed the 3-month follow-up survey.

### Clinician outcomes

Participating clinicians had a relatively high confidence rating on the PCS before the course and a slight increase in confidence with treating low back pain at 4 months post course (median difference: − 1.5, *p* < 0.001). The PABS indicated the clinicians had a combined biomedical and behavioural orientation with more of a preference for behavioral before the course. Clinicians showed significant differences in the PABS Biomedical Subscales (median difference: -4, *p*=0.005) and PABS Biopsychosocial (median difference: 2.5, *p*=0.023) which indicated clinicians were moving towards a behavioural orientation (Table 2).

**Table 3:** Patient baseline characteristics

	GLA:D® Back Group (*n*=74) baseline
Sociodemographic	
Females, *n* (%)	49 (66%)
Age, mean (SD)	55.5 (13.4)
Height	167.6 (10.5)
Weight	80.22(17.7)
No qualification	0
Vocational training	18 (24%)
Higher education < 3 years	32 (44%)
Higher education > 3 years	8 (11%)
Ordinary work, *n*=69	26 (38%)
Unemployed	0 (0%)
Rehabilitation	0
Retired	20 (29%)
Student/housewife/other, *n*=69	12 (17%)
**Clinical symptoms**	
Pain duration, *n*=72	
< 4 weeks	12 (17%)
4–12 weeks	4 (6%)
3–12 months	13 (18%)
> 1 year	43 (60%)
Previous episodes, *n*=74	
0	16 (22%)
1	10 (14%)
2–3	12 (16%)
> 3	36 (49%)
Time since treatment-initiated, *n*=71	
< 2 weeks	10 (14%)
2–4 weeks	12 (17%)
> 4 weeks	49 (69%)
No. of health care visits for present LBP, *n*=69	
1	29 (43%)
2–5	32 (47%)
6–10	4 (6%)
> 10	3 (4%)
Pain medication	*n*=82
None	41 (50%)
Over the counter	25 (30.5%)
Prescription	14 (19%)
START Back risk	
Low	12 (16%)
Medium	29 (39%)
High	33 (45%)
Sick leave last 3 months* (*n*=45)	
0 days	28 (62%)
1–14 days	12 (29%)
> 15 days	4 (9%)
EQ-5D 0-100(SD)	68.9 (18.0)

The clinician’s overall impression of the program was positive, and they were generally satisfied with the educational materials and exercise program (Table 1). Still, three clinicians were disappointed with the selection of exercises. A little more than half of the clinicians (19/35, 56%) were satisfied or very satisfied with the overall content of the exercise program, and all very satisfied/satisfied with the educational materials (Table 1).

#### Clinician interview results

Thematic analysis of content from one-on-one interviews resulted the following two themes relating to program barriers and facilitators.

#### Identified barriers

Program cost and length were often commented to be limitations by patients interested in taking the program but ultimately not committing to it.

Statements about participants financial barriers from clinicians included:

…one difficulty for people was financial… that was probably the biggest one. When recruiting, people they were really excited… That sounds amazing. That's going to work for me. And then when it came down to the financial part of it, they just couldn't do it.

Or

…so, I think we had a few factors from our end was pricing. I think we had quite a few interested but a few just couldn’t make it work in their budget.

There were some challenges for a few of the clinicians to recruit participants. Some clinicians suggested that they had difficulty with participants’ commitment to joining the program after being initially approached by clinicians and staff to join the program. Some additional reasons included timing of the classes (during the day or evening or weekends), cost of the program and committing 8 weeks for the entire program.

Statements about barriers included scheduling issues and length of the program:

…we surveyed patients and asked what would work best; either afterwork or sometime mid day…I think in general we took the mid day one because we had more people (available) from that end…but I think timing [of the program] was one of those[negative] factors…a third factor was we found some people just couldn’t commit for eight weeks

A few clinicians informed us that they would not be able to perform recruitment for various reasons including having too few patients with the required profile and having very few patients interested in the program with statements such as:

…we ran a Facebook campaign for almost two months with no response…we decided external referrals were not happening…then had a meeting to decide if we’re going to recruit candidates internally

#### Identified facilitators

Clinicians evaluated the course with high scores for each of content, usefulness and novelty (Table 1). This view was supported by one-on-one semi-structured interviews when clinicians were asked about rating the course. Representative statements included:

I thought the 2-day course flowed very well…the instructors are very knowledgeable. I felt like we had some fun doing it. I thought the course was very well done…I would rate the course as excellent

Patient recruitment: Clinicians found the 2-day clinician GLA:D® course made them more aware of which participants would benefit from the program with statements such as:

I also plan on… flagging patients who I think are moving into, or already have moved into chronic or recurrent back pain and making sure that I'm discussing this possibility [of GLA:D® Back] with them. And …immediately, my mindset is, okay…let's make sure I funnel these patients that way [into the GLA:D® program].

Clinician participants’ experience with GLA:D® Back: When asked about patients’ experiences, clinicians made statements such as:

It is a novel form of treatment that allows the patients to take care of their own issues…by using GLA:D® , it’s the exercises and knowledge that gives them [the patient participants] a long term tool that they can take home with them…showing them that movement is good and it’s not necessarily one inherent movement that is going to cause them to mess their back up…but the more movement the better…understanding that movement is good.

Group exercises: Clinicians communicated about group exercise with statements such as:

The first couple of weeks, everyone was warming up to each other in the group…now it’s a lot of fun. Everyone is really interacting with each other and enjoying each other. And it’s cool because there is now a team dynamic of learning.

Increasing capacities with exercises: As for the effect of exercise, clinicians made statement similar to these comments:

…we had one patient that had a positive straight leg raise coming into [the program] and couldn’t sit for more than 20 minutes and three quarters of the way through the program she drove 5 hours…. I asked how was sitting for that long… She just looked at me with a blank look… Then she said… you know I just realized that I didn’t have a problem.

### Patient outcomes

#### Patient adherence

After the GLA:D® Back intervention, 84% of the 78 participants reported that they attended two of the education sessions and 74% of participants attended 11–16 exercise sessions throughout the 16-session program. A small proportion of participants (*n*=2) reported that they did not receive the education portion of the program and key messages during the exercise sessions which may have a negative effect on post intervention outcomes seen after the intervention.

#### Objectively assessed physical function

From baseline to 3 months, participants (*n*=52) had a median improvement of 1 repetition on a chair stands during 30 s (*p*<0.001), a median improvement of 32 s (*p*<0.001), on the trunk flexor test (range 0–120 s), and a median improvement of 80.5 s (*p*<0.001) on the extensor endurance test (range 0–180 s) (Table 4).

**Table 4:** Patient outcome measures evaluated by Wilcoxon sign test

Variable	Pre-training median	Pre- training Q1, Q3	Post- training median	Post-training Q1, Q3	Difference in median (post-pre)	Pseudo-median	*p*
FABQ (0–24)	15	9, 18	10	3, 12	5	6.50	< 0.001
B-IPQ (0–80)	50.5	45.0, 56.0	44	39, 54	6.5	4.00	0.015
Perceived Physical Fitness (0–40)	19	16, 24	22	15, 27	3	− 1.50	0.031
Trunk Flexor Endurance (0–120 s)	39.5	19.0, 74.0	71.5	39.0, 120.0	32	− 27.00	< 0.001
Extensor endurance (0–180 s)	60	22, 120	140.5	75.0, 180.0	80.5	− 70.00	< 0.001
Sit to stand In 30 s	11	9, 13	14	12, 17	3	− 3.00	< 0.001
ODI	25	16, 34	20	10, 28	− 5	6.00	< 0.001
Back pain (0–10)	5	3, 7	3	1, 4	− 2	2.50	< 0.001
Leg pain (0–10)	2	0.5, 5.0	1	0, 3	− 1	2.00	< 0.001

*p* indicates *p* value

*Q1* first quartile, *Q3* third quartile

#### Self-reported measures

From baseline to 3 months, participants had a large median improvement of 5 (*p*<0.001), on the FABQ (range 0–24) and a small median improvement of 6.5 (*p*=0.015), on the B-IPQ surveys (Table 4).

From baseline to 3 months, participants had a minimal median improvement of − 5 (*p*<0.001) on the ODI and moderate median improvement of 2 on NPS Back pain (< 0.001) and moderate improvement of 1 on NPS Leg pain (*p*<0.001) (Table 4).

At the 3-month follow up, most patient participants (76%) were satisfied or greatly satisfied with the GLA:D® Back program and (37/50) or 74% of participants used the GLA:D® Back at home although this is not a requirement of the program (Table 5). The program was well tolerated by the participants with only 3/48 or 6.25% of participants experiencing worsening or new symptoms from the GLA:D® Back exercise sessions (Table 1). The largest change in outcome measures was seen with fear avoidance (FABQ) and the trunk flexor endurance, extensor endurance and the sit to stand in 30 s. Disability measures observed a minimal effect on ODI measures from pre- to post intervention and moderate changes were seen with leg pain and back pain (Table 4).

**Table 5:** Proportion of patients who reported they had received interventions or care outside of the GLA:D Back program in the prior month in addition to patient satisfaction results

	GLA:D Back group (*n*=53) (%)
GP	15
Chiropractic	7.5
Physiotherapy	5.6
Massage	7.5
Other	3.7
OTC medication	18.9
Prescription medication	20.8
**Number of visits in the last month**	
One time	28.6
2–5 times	57.1
6–10 times	14.3
More than 10	0
**Satisfaction with the GLA:D program**	
To a great extent	31.5
Greatly	40.7
Somewhat	22.2
To a small extent	0
Not at all	1.9
Do not know	3.7

## Discussion

This study evaluated the feasibility of implementing GLA:D® Back, a structured group education and exercise program for people with persistent or recurrent back pain in the Canadian healthcare setting. Based on our success criteria, the program was found to be feasible in this setting.

### Facilitators of GLA:D® Back adoption

GLA:D® trained clinicians were confident and motivated to implement this program which suggests that this may be a group of motivated, experienced clinicians. This group also volunteered for this study which implies that they had an affinity to this mode of treatment delivery.

Interestingly, the feasibility of the program was not heavily influenced by the current requirement that patients pay a substantial fee to participate. This requirement is a common one within this Canadian jurisdiction as most rehabilitation services are paid for out-of-pocket. Therefore, this circumstance is familiar to the Canadian public and, as a result, was not in direct competition with programs that could be accessed at no cost to patients. Still, this financial restriction would most likely prevent access to many potential participants whose demographics and case history may be significantly different from those enrolled in this study. Consequently, caution should be exerted should these results be generalized to non-participants with low back pain within the same health care region.

### Barriers of GLA:D® Back adoption

Patient recruitment was seen by clinicians as difficult especially with patients who may work in shifts or individually expressed concern about having the financial resources to participate. This financial inequity may be a driver of the observation that patients with low socioeconomic status when measured by education, past occupation, income, subjective economic situation and wealth are more predisposed to experience low back pain when compared to those with high socioeconomic status [29].

Other program barriers mentioned included that the clinic was not organizationally ready to start the program due to logistical barriers such as timing, schedules of therapists, low recruitment or associate clinicians moving to a new clinic location or other life circumstances such as being pregnant and going on maternity leave.

For those clinicians who were not successful in starting and running the program with participating patients, it is possible that they had other motivations for participating in the study. This may include expectations of more patient referrals from physicians and researchers as well as potential subsidy from the provincial healthcare system; only 10/19 (53%) were able to form a group of at least four patient participants.

### Clinician outcomes

Clinicians’ evaluation of the course was positive which may have been associated with our successful adoption rate. Also, clinicians held a moderately high orientation for a behavioral approach to care both before and after the 2-day course which also may have contributed. Small changes were observed in clinician PABS scores indicating that clinicians held a strong belief both for biomedical orientation and behavioural before the course. Congruence between clinician beliefs and the underlying principles of the GLA:D® Back program also favoured adoption. Interestingly, practitioner’s confidence was relatively unchanged from baseline to 4 months following the training course which may relate to the experience level of the clinician cohort, the practice orientation of this cohort of clinicians, the quality of the training session or other factors not measured here. As such, these results are for a fairly short period following clinician training. Longer term studies will be needed to determine if these changes are sustained.

Clinicians mentioned in the interviews that GLA:D® Back built a strong group dynamic that could be an important factor for development of self-efficacy through vicarious experience by observing other people in a similar situation. Clinicians’ also suggested that the program builds up physical capacities to match daily demands of participants’ activities. These two observations are important in this type of evidence-based program which is based on the Social Cognitive Theory (SCT) targeting the patients’ goals as SCT provides opportunities for social (group) support through instilling expectations, self-efficacy, and using observational learning and other reinforcements to achieve behavior change, while considering their individual capacity for performance [30].

### Participants outcomes

Most participants enrolled in GLA:D® Back (60%) had LBP for greater than 1 year indicating that the majority of participant reports majority of participant reports of pain duration were congruent with the inclusion criteria for the program. This is important because clinicians were appropriately targeting patients with recurrent or persistent LBP which is the condition the program was designed to address. This suggests that through intentional enrollment by clinicians, curiosity by patients or a combination of both, the majority of those in the program had persistent or recurrent LBP. Further studies should be considered to evaluate how the GLA:D® Back program may perform when used with similar populations but in different situations such as pre-surgery waitlists or post-surgical recovery.

All patient factors evaluated were done so as an exploratory exercise given the lack of a sample size calculation in this feasibility study. The B-IPQ and SF-36 did not demonstrate a significant change over time. In this case, the measurement duration may not have been enough to counteract multitude of factors that may impact a person’s beliefs and quality of life. The FABQ also showed significant improvement which suggests the education and exercise components of the program may directly address this concept. In this study, patient capacity improved as demonstrated by the performance measures. Combined, these results may motivate clinicians and patients similarly and endorse this mode of program delivery.

### Lessons learned

Feasibility may depend on clinicians properly informing patients of what to expect in the course in terms of fees, group setting and availability. Although some clinicians from this study had difficulties running the program, we found that the most motivated clinicians with a large heterogeneous LBP population were the greatest adopters of the GLA:D® Back program. Both GLA:D® Back programs in Alberta and Denmark had similar challenges with adoption. Unfortunately, as the only program outside of Denmark conducting GLA:D® Back thus far, lessons of how to improve uptake are still evolving. These lessons may include offering a wider range of program times to accommodate a range of patient schedules, emphasis placed on the potential benefits of group vs individualized programming and identifying ways to decrease out-of-pocket costs for patients to take the course.

### Study strengths and limitations

This was a feasibility study and therefore was not designed or powered to fully evaluate clinician/participants outcomes. We did not evaluate the fidelity in treatment delivery and do not know to what extent the program was delivered as intended. A future trial to evaluate the efficacy or effectiveness of GLA:D® Back is a potential consideration. This work represents the first publication of data related to an English implementation of the GLA:D® Back program which provides a basis for its use in Canada and other English-speaking jurisdictions.

### Implications for future studies

Possible directions for future studies would be to transition to a study design that evaluates the effectiveness of GLA:D® in terms of pain, disability, and self-efficacy.

## Conclusion

The English translation of the Danish GLA:D® Back program was feasible for Albertan clinicians to implement into practice in both urban and rural settings.

## Data Availability

The datasets used and/or analysed during the current study are available from the corresponding author on reasonable request.
